# Efficient Chemical Recycling of Polyester in Plastic Waste: A Heated High-Ethanol Alkaline Aqueous Process

**DOI:** 10.1021/acs.oprd.5c00386

**Published:** 2026-02-10

**Authors:** Kalliopi Elli Pavlopoulou, Vincenzo Ianniello, Kateřina Hrůzová, Theo A. Tervoort, Heiko Lange, Ulrika Rova, Paul Christakopoulos, Leonidas Matsakas

**Affiliations:** † Biochemical Process Engineering, Dept. of Civil, Environmental and Natural Resources Engineering, 5185 Luleå University of Technology, SE-971 87 Luleå, Sweden; ‡ Department of Materials, 27219 ETH Zurich, Vladimir-Prelog-Weg 5, 8093 Zurich, Switzerland; § Department of Earth and Environmental Sciences, 9305 University of Milano-Bicocca, Piazza della Scienza 1, 20126 Milan, Italy; ∥ NBFC−National Biodiversity Future Center, 90133 Palermo, Italy

**Keywords:** polyester, PET bottles, heated high-ethanol alkaline process, hydrolysis, plastic recycling

## Abstract

Plastic waste, especially from packaging, poses major recycling challenges due to the presence of mixed polymers, which often result in inconsistent blends that are unsuitable for reuse in food-grade applications. Chemical recycling, particularly alkaline hydrolysis, offers a promising solution in the case of chemically reactive polymers, such as polyesters, with poly­(ethylene terephthalate) (PET) being one of the dominant plastics suitable for both mechanical and chemical recycling. Mechanical recycling is currently used for the largest part of PET recycling, due to the fact that turning the polymer back into its monomeric building blocks requires catalysts, elevated temperatures, or prolonged reaction times. This study presents a recently developed Heated High-Ethanol Alkaline Aqueous (HHeAA) process that enables efficient, catalyst-free PET hydrolysis under milder conditions. Nearly complete hydrolysis was achieved within just 20 min at 90 °C using a loading of 0.624 g of NaOH/g of PET. The process was successfully scaled up with commercial PET bottles, achieving full hydrolysis while significantly reducing the liquid-to-solid ratio from 20 to just 5 L/kg. These results highlight the industrial potential of the HHeAA method as a more sustainable and energy-efficient alternative for PET recycling and chemical reuse and in turn reduced environmental impact.

## Graphical abstract

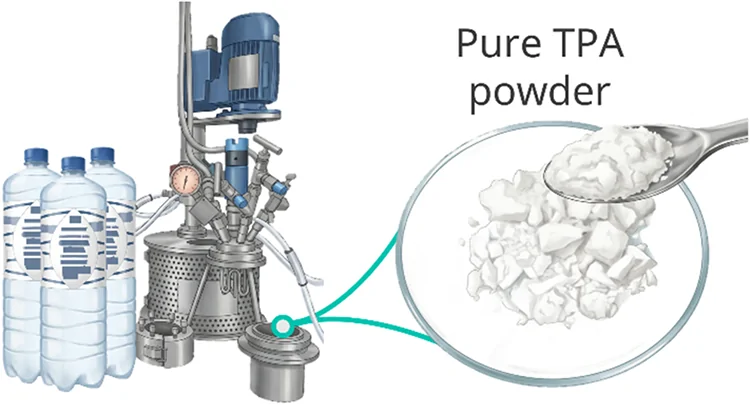

## 1 Introduction

The 1950s marked a turning point for modern society, as plastic materials began to permeate everyday life in the form of textiles, fashion, toys, and household goods, and plastics quickly gained popularity due to their affordability, versatility, and durability.^1^ Polyethylene (PE) bags emerged, while iconic brands like Mattel and LEGO launched widely popular plastic-based toys. By 1970s, poly­(ethylene terephthalate) (PET) beverage bottles were introduced, followed by expanding use in technological applications such as mobile phones, watches, and components for automotive and aerospace industries. By 1976, plastics had become the most widely used materials globally.^1^

While plastics have transformed daily life, their durability has become a significant environmental challenge. PET, a semicrystalline polyester, is widely used for numerous applications due to its mechanical and thermal properties. The physical properties of PET arise from its semicrystalline nature, composed of both crystalline and amorphous regions.^2^ During cooling from the melt, crystalline regions form spherulitic structures through nucleation and crystal growth.^3^ The degree of crystallinity is strongly influenced by thermal history, particularly the cooling rate, a key factor in defining the final microstructure of the material.^3–6^ By controlling the crystallization rate, it is possible to obtain a material with a higher proportion of ordered crystalline structures compared to amorphous regions. Since the crystal growth rate is higher than the nucleation rate, slow cooling allows more extensive crystal growth to form, resulting in higher degree of order, while rapid cooling restricts crystal formation and promotes an amorphous structure. Extremely fast cooling rates can even suppress nucleation completely, yielding fully amorphous PET.^7,8^

Crystallinity can also be controlled by the processing methods. Melt processing with controlled cooling rates is one approach. Alternatively, solvent casting, where the polymer is first dissolved in a suitable solvent followed by slow evaporation at room temperature, can yield materials with varied morphologies depending on solvent quality, polymer concentration, and evaporation rate.^9–13^

PET can be found in a range of everyday life products, from textiles to packaging and even in disposable products. It is ideal for food packaging and bottles due to its transparency, chemical resistance, recyclability, and gas barrier properties.^14^ Furthermore, it is inert and does not leave a taste in the containing liquids.^15^ However, the resistance of PET to environmental and biological degradation has contributed to its accumulation in ecosystems, underscoring the urgency for effective recycling methods.^14,16^ The primary recycling approaches for PET include mechanical and chemical recycling, each with distinct advantages and limitations.

Mechanical recycling remains the dominant method for PET reuse, involving processes such as sorting, washing, drying, and remelting to produce recycled PET (rPET). While cost-effective and relatively low in environmental impact, mechanical recycling has significant limitations. The main among these is downcycling, where the recycled product is of lower quality and utility compared to the original.^16,17^ Although PET is the most recycled plastic (recycling percentage reaches 30%), it is susceptible to downgrading through thermal and hydrolytic mechanisms during recycling cycles leading to reduced mechanical strength and processability.^17,18^ This thermomechanical degradation restricts the number of recycling cycles before the material becomes unsuitable for further use.^17^

Contamination with, for example adhesives, and pigments, can compromise rPET quality.^19^ Even trace contaminants can cause molecular weight reduction and discoloration of the final product, leading to a polymer with unpredictable properties.^20^ This limits the suitability of the recycled material for high-value applications, such as food packaging, which have specific regulations and standards. As a result, a large portion (50–70%) of rPET is diverted to lower-value applications, such as fibers in carpeting, instead of the original packaging application.^18^ Additionally, degradation products like acetaldehyde raise concerns for food-contact safety.^17^ These drawbacks lead to a limited demand and low market value of recycled materials due to varying quality.^21–24^

Chemical recycling offers a promising alternative by depolymerizing PET into its original monomers, enabling true circularity. Main methods include glycolysis, hydrolysis, methanolysis, and ammonolysis, each using different reagents to break down PET into reusable monomers.^17,25^ The routes are listed in Figure 1.

**Figure 1:**
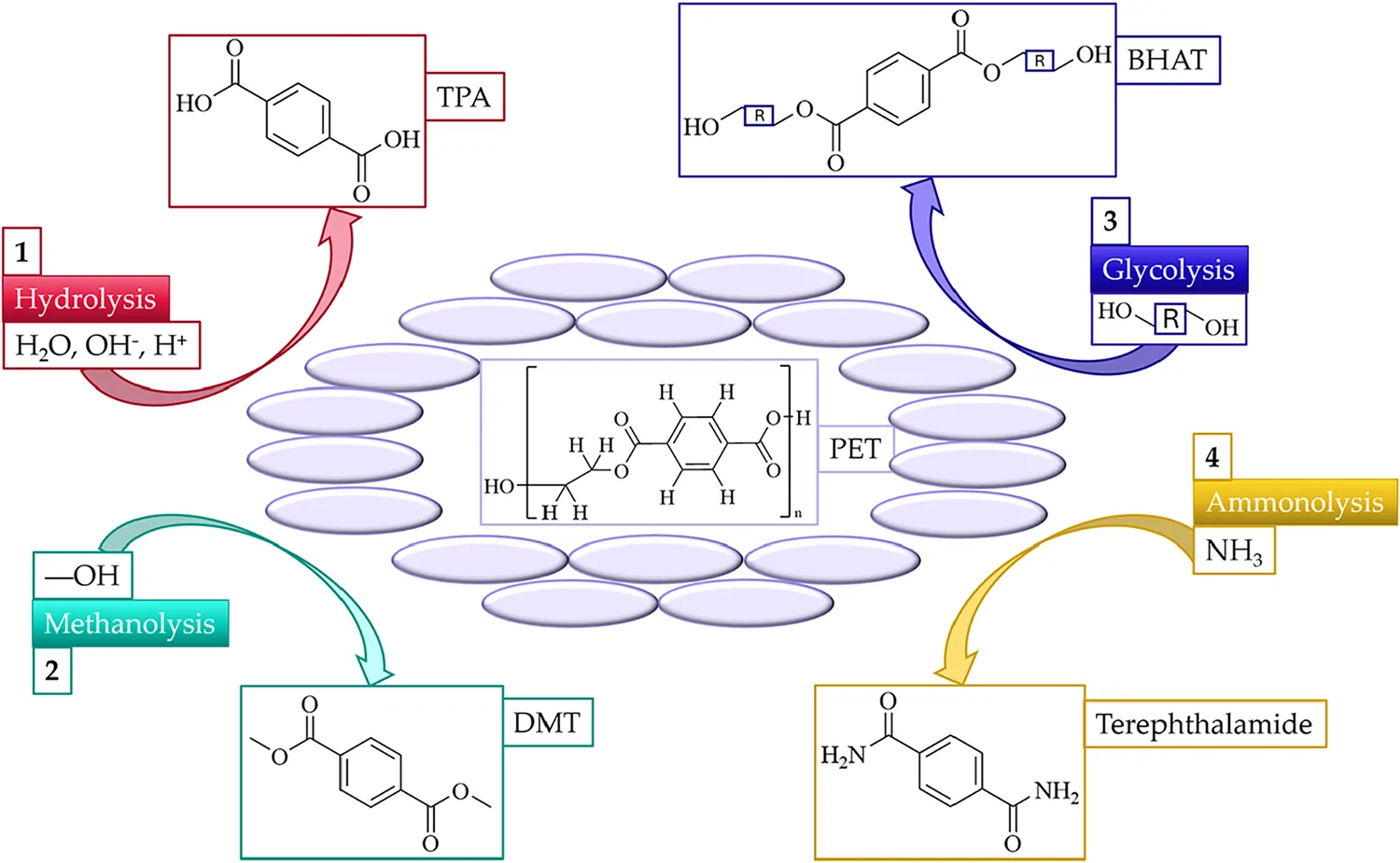
Depiction of the four primary depolymerization reactions of PET and their main products: (1) hydrolysis, yielding terephthalic acid (TPA); (2) Methanolysis, yielding dimethyl terephthalate (DMT); (3) Glycolysis, yielding bis­(hydroxyalkyl)­terephthalate (BHAT), where R represents the alkyl chain corresponding to the glycol reagent used; and (4) Ammonolysis, yielding terephthalamide. All of these reactions also generate ethylene glycol as a subproduct. Modified from Pavlopoulou et al.^26^ Copyright 2024 by the authors, published by MDPI. Licensed under the provisions of the Creative Common Attribution CC-BY 4.0 License (https://creativecommons.org/licenses/by/4.0/).

Hydrolysis yields high-purity terephthalic acid (TPA) and ethylene glycol (EG) but typically requires elevated temperatures (200–250 °C) and high pressures, making it energy-intensive.^25^ Glycolysis, which produces bis­(hydroxyethyl) terephthalate (BHET), is used due to its relatively mild conditions and industrial adaptability when combined with catalysts and other methods, such as microwave heating, thus reducing significantly the reaction time, retaining high-temperature demands nonetheless (180–300 °C).^27^ Methanolysis breaks PET into dimethyl terephthalate (DMT) and EG, but the shift in PET production from DMT to TPA has reduced its viability.^27^ Ammonolysis is less commercially developed and primarily explored for modifying PET fibers.^25,27^

Despite the advantages of monomer recovery and upcycling, chemical recycling faces obstacles, such as high operational costs, high energy demands, and complex purification steps that hinder widespread adoption. Nonetheless, chemical recycling aligns better with long-term sustainability goals than mechanical recycling and offers potential if key technological and regulatory gaps are addressed.^17,25^

Hydrolysis can be performed in neutral, acidic, or alkaline environments. Acidic hydrolysis becomes highly costly since it is needed to recycle large volumes of concentrated acids and purify the products from them, while the temperature requirements can range from 90 to 150 °C depending on the concentration of the acids.^25^ Neutral hydrolysis demands higher temperatures compared to the other options, since it usually runs at 200–300 °C.^25^ On the other hand, alkaline hydrolysis takes place at a range of temperatures between 60–200 °C in the presence of high sodium hydroxide (NaOH) concentrations that vary between 2–20% w/v based on latest studies.^21–24^ This reaction hydrolyzes the ester bonds in PET molecules, resulting in its two components: EG and TPA—the latter in the form of disodium terephthalate (Na_2_TPA).^26^ The Na_2_TPA is then treated with an acid, commonly sulfuric acid, to convert it to TPA, which is water insoluble and is recovered at high purity.^22^ The mechanism of alkaline hydrolysis is illustrated in Figure 2.

**Figure 2:**
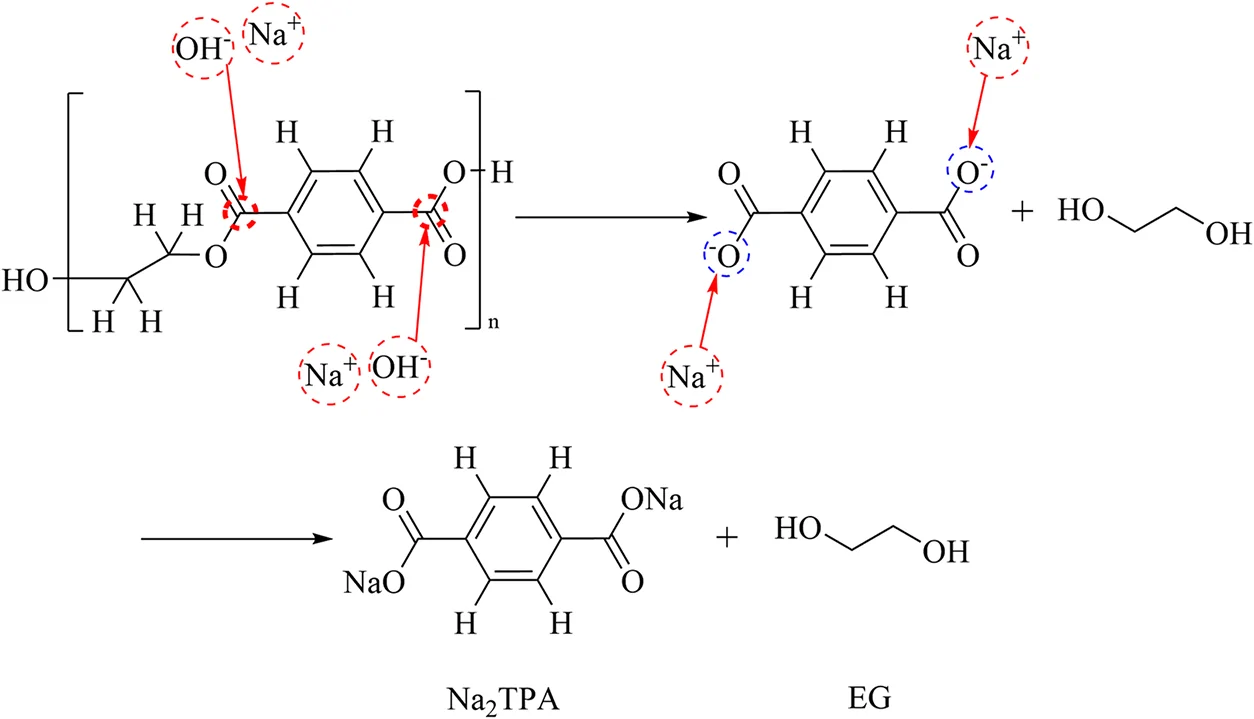
Illustration of the PET alkaline hydrolysis reaction.

From a mechanistic perspective, the reaction proceeds via a nucleophilic acyl substitution, where the hydroxide ion attacks the carbonyl carbon of the ester group. This leads to the formation of a tetrahedral intermediate, followed by the release of the alkoxide and an acid–base proton transfer to yield the stable terephthalate salt (Na_2_TPA) and ethylene glycol.^28,29^

A promising recent advancement is the Heated High-Ethanol Alkaline Aqueous (HHeAA) process, initially developed by Pavlopoulou et al. for the treatment of polycotton.^26^ This technique enables efficient PET hydrolysis under milder conditions, lower temperatures, reduced NaOH concentrations, and shorter reaction times, without the use of catalysts.

The aim of this study is to enhance the recently developed HHeAA process for the effective recycling of PET and to extend its applicability to a broader range of waste plastics. Key process parameters are systematically investigated and optimized to maximize hydrolysis efficiency while minimizing chemical waste and to perform the process at less energy demanding conditions that are typically used in alkaline hydrolysis, addressing limitations of conventional alkaline hydrolysis methods.^23,30^ Additionally, the study explored the feasibility of applying the HHeAA process for the treatment of plastic bottles, highlighting its potential for plastic recycling and paving the way for its application to other waste packaging materials.

## 2 Experimental Section

### 2.1 Heated High-Ethanol Alkaline Aqueous Treatment of Pristine PET

#### 2.1.1 Materials

Commercial PET chips (WK801, PET bottle-grade; Sabic, Riyadh, Saudi Arabia) were kindly provided by Sabic and used as the feedstock for the experiments. The material properties, as supplied by the manufacturer, are summarized in Table 1. The chips were cryomilled using a Freezer Mill 6770 (Spex Sample Prep, Metuchen, NJ) to obtain a fine powder. Cryomilling was performed in three cycles, each consisting of 2 min of grinding at 15 cps followed by a 1 min rest. The resulting powder was dried under a vacuum at 50 °C overnight and subsequently sieved through a 1 mm mesh. For each experiment, a constant PET powder mass of 5 g was used.

**Table 1:** Main Parameters of the Used PET Provided by the Company

property	typical value	limit
intrinsic viscosity	0.8 dL/g	±0.02
acetaldehyde content	1 ppm	max
moisture content	0.4 wt %	max

#### 2.1.2 Design of Experiments and the Hydrolysis Procedure

A Design of Experiments (DoE) approach was employed to investigate the effects of the ethanol content, sodium hydroxide concentration, and temperature on PET hydrolysis. These factors were selected for initial optimization to minimize chemical consumption and energy demands in terms of process temperature before the reaction duration is optimized. The study used Design-Expert v13 software (StatEase, Minneapolis, MN) with a Box-Behnken 3 × 3 factorial design, incorporating three numeric factors and three center points per block (Table 2). The response variable was the hydrolysis yield (%), and a total of 15 experimental runs were generated, detailed in Table 3.

**Table 2:** Set Factors, Their Units, and Limits

	name	units	low	high
A [numeric]	EtOH	% (v/v)	70	90
B [numeric]	g NaOH/g PET		0.416	1.248
C [numeric]	temperature	°C	70	110

**Table 3:** Generated Set of Experimental Conditions^a^

		factor 1	factor 2	factor 3
std	run	A: EtOH (% (v/v))	B: g NaOH/g PET	C: temperature (°C)
1	14	70	0.416	90
2	6	90	0.416	90
3	8	70	1.248	90
4	15	90	1.248	90
5	10	70	0.832	70
6	13	90	0.832	70
7	7	70	0.832	110
8	3	90	0.832	110
9	9	80	0.416	70
10	1	80	1.248	70
11	12	80	0.416	110
12	4	80	1.248	110
13	2	80	0.832	90
14	5	80	0.832	90
15	11	80	0.832	90

a Note: g NaOH/g PET to NaOH/PET molar ratio: 0.416 = 2:1; 0.832 = 4:1; 1.248 = 6:1.

During HHeAA, the reaction mixture consisted of distilled water, absolute ethanol (≥99.8% (v/v); VWR, Radnor), and sodium hydroxide (Sigma-Aldrich, St. Louis, MO). The NaOH concentration was expressed as g NaOH/g PET, and these specific concentrations were selected based on the molar rates of the chemical reaction between NaOH and PET, where 0.416 g NaOH/g PET represents a molar ratio of NaOH/PET equal to 2/1, which is the stoichiometric requirement for the reaction. The milled PET (5g) was mixed with 100 mL of ethanol to water solution with a concentration of 70, 80, and 90% (v/v) ethanol to achieve a liquid-to-solid ratio (LSR) of 20, containing the corresponding NaOH quantity.

Each reaction was conducted in a 250 mL stirred pressure reactor autoclave system (Amar Equipment, Mumbai, India). The PET powder and prepared solvent mixture were loaded into the reactor, heated to the target temperature, and maintained at that temperature for 60 min. After reaction completion, the mixture was cooled to ∼25 °C, transferred to a beaker, and diluted with 167 mL of distilled water to reduce NaOH and EtOH concentrations and fully dissolve the produced Na_2_TPA.

The diluted mixture was then vacuum-filtered, and the filtrate was kept for further analysis. The preweighted filter paper containing the unreacted PET was rinsed with an additional 167 mL of distilled water, and this wash-filtrate was also collected for analysis and is hereafter referred to as “wash.” Finally, the filter paper was rinsed with additional water to remove any remaining soluble impurities, dried in an oven at 60 °C overnight, followed by the gravimetric determination of residual PET. The degree of hydrolysis, *i.e*., the hydrolysis yield was then calculated using eq 1

1 $$PET\,\;\;hydrolysis\;\left(\%\right)\;=\;\left(1-\frac{g_{recovered\,\;PET}}{5\;g}\right)\times 100$$

Following the completion of all of the experimental runs, the response data were analyzed *via* ANOVA to determine the best-fitting model. Optimization was subsequently performed graphically to determine the ideal reaction conditions.

#### 2.1.3 Effect of Reaction Time on PET Hydrolysis

To investigate the influence of reaction time on PET hydrolysis, four sets of conditions were selected based on the initial experimental design. Each condition was tested at three different time intervals: 20, 40, and 60 min using an LSR of 20 (Table 4).

**Table 4:** Set of Conditions for the Time Effect Study

parameter	set 1	set 2	set 3	set 4
EtOH (% v/v)	90	90	90	90
g NaOH/g PET	0.624	0.624	0.832	0.832
temperature (°C)	80	90	80	90
time (min)	20, 40, 60	20, 40, 60	20, 40, 60	20, 40, 60

The procedure was optimized to enable an effective separation of the reaction products. After completion, the resulting slurry was filtered to separate the ethanol-rich filtrate (EtOH Filtrate) from the solid Na_2_TPA product. Two subsequent washing steps were performed: (1) a 200 mL distilled water wash to dissolve the solid Na_2_TPA (Wash 1), and (2) an additional 200 mL distilled water rinse to remove any remaining Na_2_TPA (Wash 2). The filter paper containing any unreacted PET was then dried and weighed to determine the extent of the hydrolysis.

### 2.2 Heated High-Ethanol Alkaline Aqueous Treatment of PET Bottles

#### 2.2.1 Effect of Reaction Time

To validate the hydrolysis process using PET waste based on commercialized PET products, commercially available, uncolored PET bottles used for noncarbonated water were sourced from a local supermarket. After the caps and labels were removed, the bottles were cut into square pieces measuring approximately 3 × 3 cm. In each experiment, 5 g of PET bottle pieces were used.

Four experimental conditions were studied, combining two temperatures, 90 and 80 °C, with two mass ratios of NaOH/PET, 0.624 and 0.832. Each condition was tested at three different reaction times, *i.e*., 20, 40, and 60 min. The downstream separation procedure described in Section 2.1.3 was followed. Hydrolysis yields were calculated based on the mass of unreacted PET relative to the initial PET mass.

#### 2.2.2 Liquid-to-Solid Ratio Optimization

To explore the scalability of the HHeAA process, the impact of reduced liquid volumes was assessed by performing experiments using larger PET quantities. Bottle-derived PET was cut into rectangular pieces (0.5 × 0.3 cm), and experiments were carried out using 11.43 and 16.00 g of PET. The PET pieces were mixed with 80 mL of ethanol–water solution (90% (v/v) EtOH), containing 0.624 g NaOH/g PET. Due to increased PET content, solid NaOH pellets were directly added to the reaction vessel along with EtOH and water to ensure complete dissolution during reaction. These setups corresponded to reduced LSRs from 20 to 7 and 5, respectively, calculated using the volume of aqueous EtOH reaction solution and mass of PET solids. All reactions were carried out at 90 °C for 20 min. For the LSR 5, additional experiments were conducted at longer durations, *i.e*., 30, 40, and 60 min, to investigate the impact of reaction time in more concentrated systems.

After treatment, the reaction mixture was cooled to room temperature and then mixed with 400 mL of distilled water to dissolve the resulting paste of Na_2_TPA. This solution was filtered through a cellulose filter (Qualitative 401, VWR, Radnor, PA) and the filtrate containing the Na_2_TPA and EG was collected and stored in plastic bottles at room temperature. The remaining unreacted PET solids on the filter paper were washed with distilled water to remove residual traces of Na_2_TPA or NaOH, then dried, and weighed to determine the hydrolysis yield.

Mass balances were performed to assess the recovery of the key monomers: EG and TPA. The EG content in the liquid fraction was quantified using high-performance liquid chromatography (HPLC) (refer to Section 2.3.2). TPA was recovered by acidifying the Na_2_TPA solution to pH 2 using concentrated sulfuric acid (98% H_2_SO_4_, Sigma-Aldrich, St. Louis, MO). The resulting precipitate was filtered, dried, and weighed. Duplicate determinations were performed on 100 mL aliquots. The purity of recovered TPA was evaluated by HPLC, proton nuclear magnetic resonance (^1^H NMR) and ash content analysis (refer to Sections 2.3.1, 2.3.2, and 2.3.3, respectively). A schematic overview of the process is provided in Figure 3.

**Figure 3:**
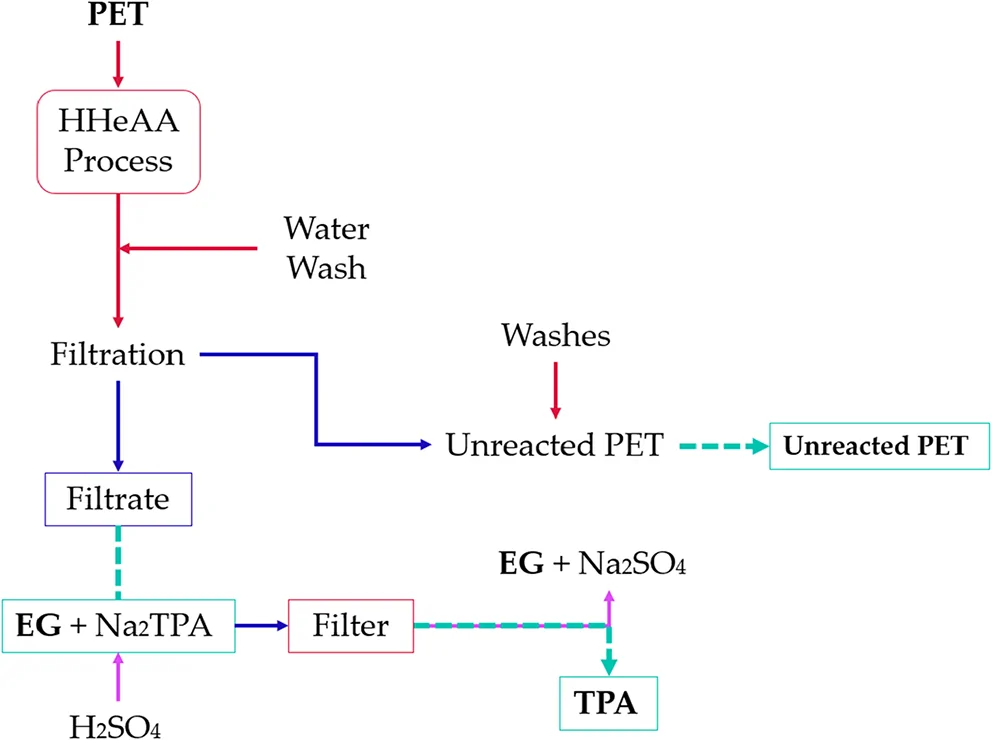
Flow diagram of the scaled experiments.

### 2.3 Analytics

#### 2.3.1 Nuclear Magnetic Resonance (NMR) Spectroscopy

The purity of recovered TPA was assessed using ^1^H NMR spectroscopy. A 60 MHz X-pulse Benchtop NMR spectrometer (Oxford Instruments, Abingdon-on-Thames, UK) was employed for the analysis. Approximately 40 mg of TPA was dissolved in 600 μL of DMSO-*d*_6_ (Cambridge Isotope Laboratories, Tewksbury) and transferred to a standard 5 mm NMR tube for analysis. Purity was evaluated on the basis of the ratio between the characteristic TPA aromatic proton signal at 8.0 ppm and the signal caused by the presence of EG between 3.5 and 4 ppm.

#### 2.3.2 High-Performance Liquid Chromatography (HPLC)

Quantification of EG was performed using an HPLC equipped with a refractive index detector (PerkinElmer, Waltham, MA). A BioRad Aminex HPX-87H-column (Hercules, CA) was used, maintained at 65 °C. The mobile phase consisted of 5 mm H_2_SO_4_ at a flow rate of 0.6 mL/min. Prior to injection, samples were acidified to pH 3 and filtered through 0.2 μm hydrophilic syringe filters to remove any residual Na_2_TPA.

To assess the presence of physically entrapped EG within the recovered TPA crystals, we performed a water extraction assay. An accurately measured amount of ∼0.35 g of TPA powder was suspended in 3 mL of distilled water and incubated overnight at 25 °C under agitation. The supernatant was then filtered and analyzed for EG content by using the method described above.

The purity of the recovered TPA was also analyzed using the same HPLC system equipped with a Macherey-Nagel NUCLEOSIL 100-5 C18 HD column (Dueren, Germany) maintained at 40 °C. This method was employed to separate TPA from potential incomplete hydrolysis products containing chemically bound EG, *i.e.*, BHET and mono­(2-hydroxyethyl) terephthalate (MHET). The mobile phase was an isocratic mixture of solvent A (milli-q water with 0.1% (v/v) trifluoroacetic acid; VWR, Radnor, PA) and solvent B (acetonitrile; Merck, Darmstadt, Germany) in an 80:20 (v/v) ratio with a flow rate of 1 mL/min. Solid TPA samples were dissolved in DMSO (Sigma-Aldrich, St. Louis, MO), diluted with the mobile phase, and filtered through 0.2 μm hydrophilic syringe filters prior to injection. The Agilent G1314B variable wavelength detector (Santa Clara, CA) was used for the analysis measuring at 242 nm.

The following compounds were used as a standard for calibrations: TPA (Sigma-Aldrich, St. Louis, MO), BHET (Sigma-Aldrich, St. Louis, MO), and EG (VWR, Radnor, PA).

#### 2.3.3 Ash Content Analysis

To assess the inorganic impurity level, recovered TPA samples from the LSR effect experiments were subjected to gravimetric ash analysis. Samples were ashed at 550 °C for 3 h to determine inorganic ash content gravimetrically.

#### 2.3.4 Scanning Electron Microscopy (SEM)

To examine the morphology of PET bottles (untreated and unreacted fragments after HHeAA), an EI Magellan 400 field-emission XHR-SEM (Thermo Fisher Scientific, Waltham, MA) scanning electron microscope (SEM) was used. Before the analysis, the samples were put on conductive carbon tape. A low accelerating voltage of 2 kV and a beam current of 3.1 pA were used to take pictures.

#### 2.3.5 Differential Scanning Calorimetry (DSC)

Thermal analysis of PET samples was conducted using a DSC 2500 differential scanning calorimeter (TA Instrument, New Castle, DE). Each sample underwent two consecutive heating and cooling cycles from 25 to 300 °C at a constant rate of 5 °C/min.

The degree of crystallinity (*X*_c_) was calculated from the first heating cycle using eq 2

2 $$X_{c}\,\;=\;\frac{{\Delta H}_{m}-{\Delta H}_{c}}{{\Delta H}_{f}^{0}}$$

where Δ*H*_m_ is the enthalpy of fusion obtained from the endothermic peak, Δ*H*_c_ is the enthalpy of crystallization from the exothermic peak due to recrystallization, and Δ*H*_f_^0^ is the standard heat of fusion for 100% crystalline PET, taken as 140 J/g.^31,32^

## 3 Results and Discussion

### 3.1 Experiments with Pristine PET

#### 3.1.1 Experimental Design for Process Optimization

To identify mild, in terms of processing temperature and NaOH loading, yet effective conditions for PET hydrolysis, we employed a design of experiments (DoE) approach to explore the influence of important process parameters: ethanol concentration, mass ratio between PET and NaOH, and reaction temperature. The goal was to determine suitable ranges of those parameters that maximize hydrolysis yield while minimizing chemical use and temperature. As such, the experimental matrix included a broad range of parameter combinations (Table 5).

**Table 5:** Levels of Each Variable and the Corresponding Hydrolysis Percentage Obtained from the Box-Behnken Design

		factor 1	factor 2	factor 3	response 1
std	run	A: EtOH (% (v/v))	B: g NaOH/g PET (−)	C: temperature (°C)	hydrolysis (%)
5	10	70	0.832	70	78.91
1	14	70	0.416	90	90.89
3	8	70	1.248	90	99.64
7	7	70	0.832	110	99.50
9	9	80	0.416	70	68.56
10	1	80	1.248	70	96.64
13	2	80	0.832	90	99.10
14	5	80	0.832	90	99.10
15	11	80	0.832	90	97.50
11	12	80	0.416	110	94.32
12	4	80	1.248	110	99.28
6	13	90	0.832	70	87.50
2	6	90	0.416	90	96.11
4	15	90	1.248	90	99.29
8	3	90	0.832	110	99.29

As shown in Table 5, hydrolysis yields were generally very high across almost all tested combinations, with only two runs respectively falling below 80% yield were employing 70% (v/v) EtOH with 0.832 g NaOH/g PET at 70 °C (Run 10) and 80% (v/v) EtOH with 0.413 g NaOH/g PET at 70 °C (Run 9). These results highlight the significant influence of the temperature and NaOH concentration on reaction performance. Notably, even under relatively mild conditions, *i.e*., 70% (v/v) EtOH, 0.416 g NaOH/g PET, and 90 °C, the yield remained high with 90.9% (Run 14). The analysis of variance (ANOVA) results are presented in Table 6.

**Table 6:** ANOVA for the Hydrolysis Percentage According to the Response Surface Reduced Quadratic Model

source	sum of squares	df	mean square	*F*-value	*p*-value	
model	1078.35	7	154.05	15.65	0.0009	significant
A- EtOH	21.95	1	21.95	2.23	0.1790	
B- g NaOH/g PET	252.79	1	252.79	25.68	0.0015	
C- temperature	461.78	1	461.78	46.91	0.0002	
AC	19.36	1	19.36	1.97	0.2035	
BC	133.63	1	133.63	13.58	0.0078	
B^2^	12.35	1	12.35	1.25	0.2996	
C^2^	182.31	1	182.31	18.52	0.0036	
residual	68.90	7	9.84			
lack of fit	67.20	5	13.44	15.75	0.0608	not significant
pure error	1.71	2	0.8533			
cor total	1147.25	14				
*R* ^2^ = 0.9399	*R* ^2^ (Adj) = 0.8799					

The fitted model showed a strong statistical significance with an *F*-value of 15.65 and a low probability (0.09%) of such a result arising from random noise. The coefficient of determination (*R* ^2^ = 0.9399) for the model indicates that 93.99% of the variability in hydrolysis yield can be explained by the model predictors. The adjusted *R* ^2^ of 0.8799 suggests minimal overfitting and confirms the robustness of the model. The lack-of-fit *F*-value of 15.75, corresponding to a 6.08% chance of being caused by random variation, further supports the statistical relevance of the model.

The p-values associated with the model terms indicated that several variables and interactions significantly affect the hydrolysis efficiency. Specifically, the mass ratio between PET and NaOH (B), temperature (C), their interaction (BC), and the quadratic temperature term (C^2^) all had p-values well below 0.05. The significance of the quadratic temperature term C^2^ suggests a nonlinear relationship between temperature and yield, implying an optimal temperature range beyond which further increases may not improve performance. Furthermore, the BC interaction term (*p* = 0.0078) had a much stronger effect than the AC term (*p* = 0.2035), indicating a synergistic relationship between the NaOH concentration and temperature. Ethanol content (A) had a comparatively higher p-value (*p* = 0.179), suggesting a less significant role in the reaction outcome compared to NaOH and temperature within the tested design space. However, it is critical to distinguish between the statistical significance in the regression model and the practical engineering importance. While the yield is resilient to minor fluctuations in ethanol concentration, as reported for similar molecules in other studies,^33^ the presence of ethanol is a fundamental driver enabling the milder reaction conditions and high efficiency observed in this study.

From a mechanistic perspective, ethanol facilitates depolymerization *via* two key synergistic pathways: enhanced polymer accessibility and product solubility control.

First, ethanol acts as an effective plasticizer for the PET matrix. Small organic molecules such as ethanol are known to be able to penetrate the amorphous regions of PET, increasing free volume and significantly reducing the glass transition temperature of *T*_g_ of the solvent polymer.^34^ Under purely aqueous conditions, the rigid glassy state of PET limits the diffusion of the hydroxide nucleophile.^24^ In the ethanol–water cosolvent system, the decrease of *T*_g_ allows the polymer chains to transition into a more mobile, rubber state at lower temperatures.^35^ This increased segmental mobility, coupled with solvent-induced swelling, creates expanded diffusion channels that drastically accelerate the mass transfer of hydroxide ions to the internal ester linkages,^35,36^ thus explaining the rapid kinetics achieved without the harsh temperatures required by neutral or purely aqueous hydrolysis.

Second, ethanol is engineering-essential for downstream product recovery. Disodium terephthalate exhibits markedly lower solubility in ethanol-rich mixtures compared to pure water.^37^ This solubility difference promotes the spontaneous precipitation of Na_2_TPA as a distinct solid phase. Furthermore, ethanol recovery is a well-established industrial process, and this enables its reuse in subsequent treatments, thus minimizing the use of fresh ethanol. Therefore, while the specific concentration of ethanol acts as a statistically nonsignificant parameter in the DoE model, its physicochemical role in plasticizing the polymer and precipitating the product is the prerequisite for the process’s viability.

To achieve high yields under mild conditions, a hydrolysis efficiency of >95% is desirable with reduced chemical input and energy. Reducing the reaction temperature lowers energy consumption and pressure buildup, simplifying the reactor design and enhancing process safety. Additionally, minimizing NaOH usage minimizes chemical consumption.

It is also important to note the significant effect of the glass transition temperature (*T*_g_) on the hydrolysis reaction yield. PET has a *T*_g_ in the range of 75–80 °C. As the polymer’s temperature approaches and exceeds its *T*_g_, the amorphous regions undergo a transition from a rigid, glassy state to a more flexible, rubbery state, increasing segmental mobility of the polymer chains.^38^ This enhanced mobility is crucial as it improves the diffusion and accessibility of water molecules and hydroxide ions to the ester linkages within the polymer matrix, thereby significantly facilitating the hydrolysis process.^38^ Consequently, conducting hydrolysis at temperatures above *T*_g_, *i.e*., at like 80–90 °C, ensures sufficiently mobile polymer chains to effectively allow the penetration of reactants into the matrix, maximizing reaction kinetics and overall yield.

As shown in Figure 4, increasing ethanol concentration under constant NaOH load allows for lower operating temperatures while maintaining high hydrolysis yield. Additionally, a small increase in the NaOH concentration can further reduce the required temperature while maintaining high yields (Figure 5).

**Figure 4:**
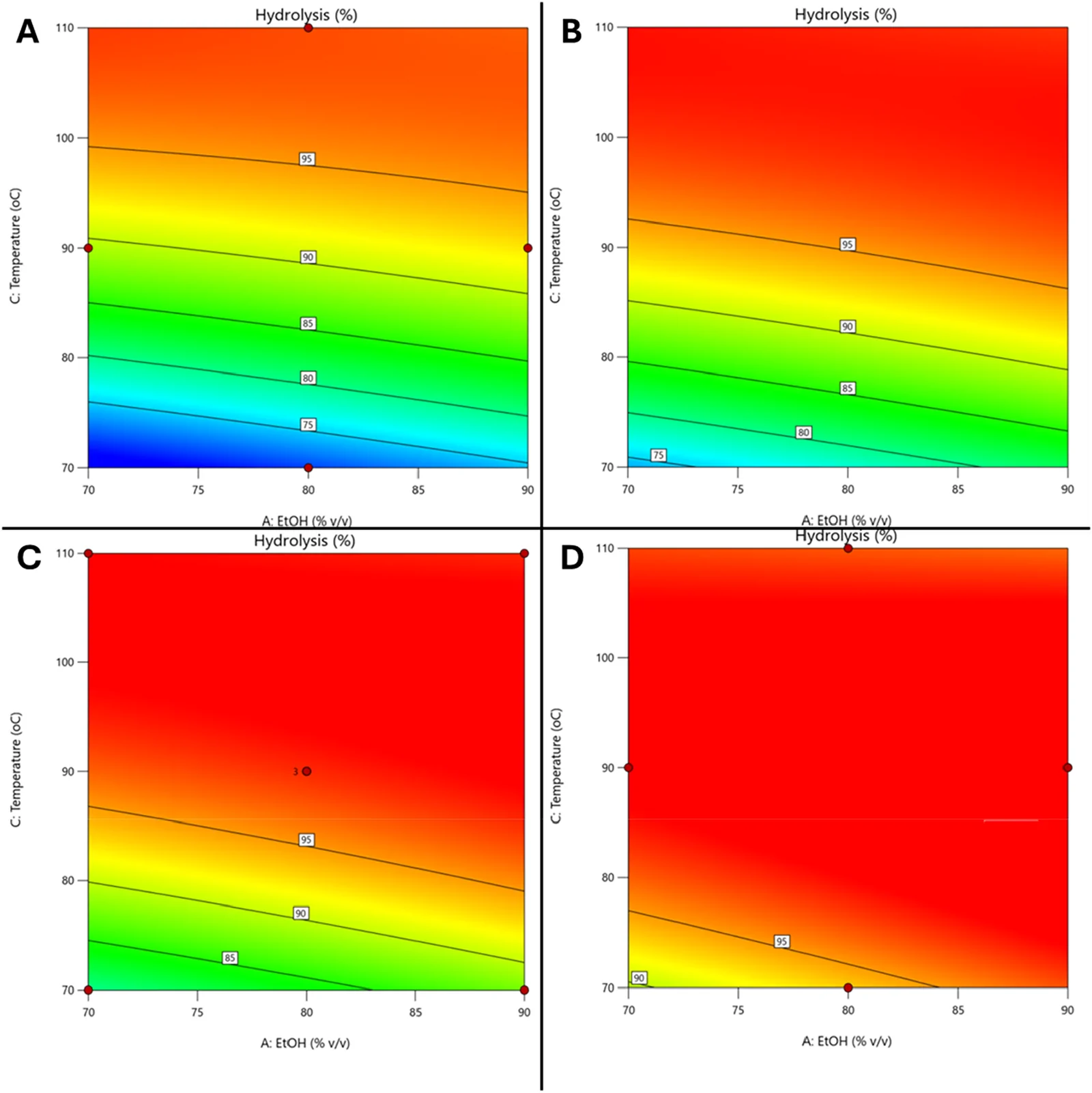
Contours for temperature and EtOH content with a varying g NaOH/g PET ratio. (A) 0.416, (B) 0.624, (C) 0.832, and (D) 1.248 g.

**Figure 5:**
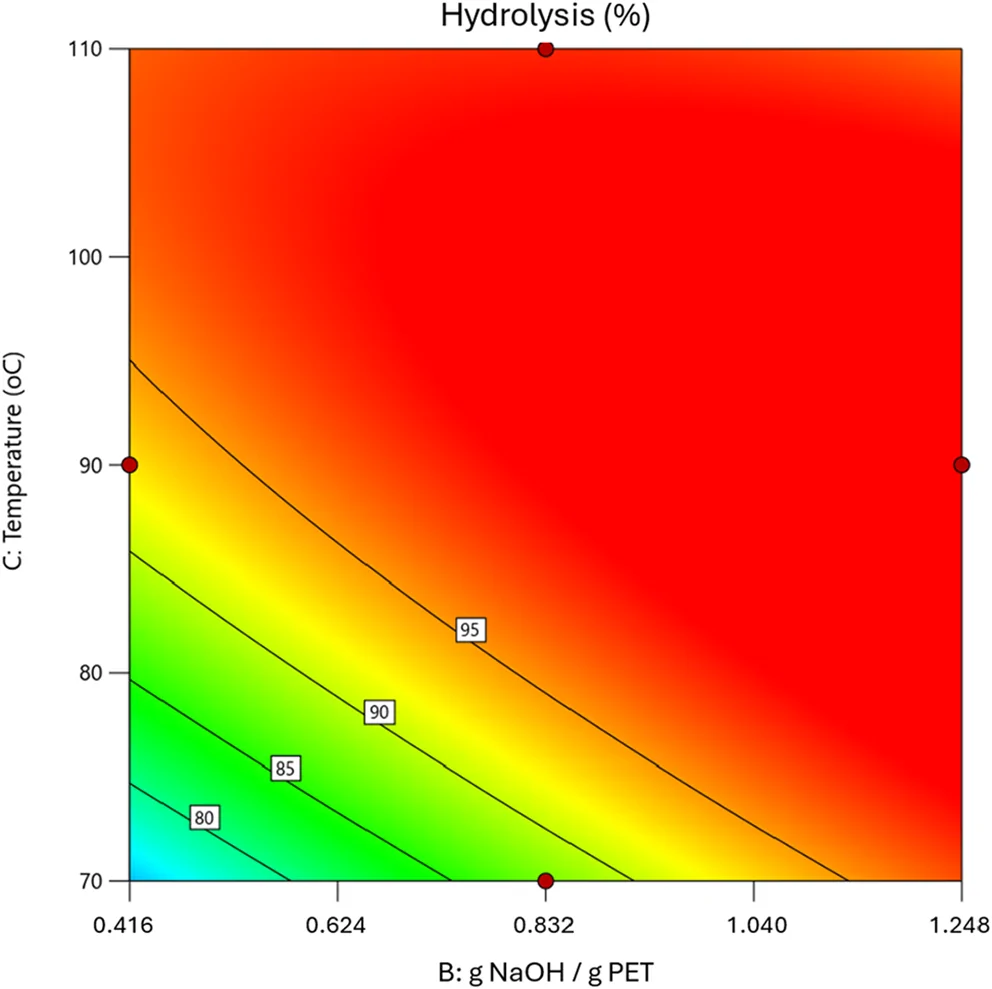
Contour for temperature and the NaOH:PET ratio for 90% (v/v) EtOH content.

For instance, at 90% (v/v) EtOH with 0.832 g of NaOH/g of PET ratio, hydrolysis yields exceed 95% even at 80 °C. While a higher NaOH loading of 1.248 g of NaOH/g of PET also achieves high yields, it represents an excessive use of chemicals. Conversely, a reduced NaOH loading of 0.416 g NaOH/g PET demands higher temperatures, nearly 100 °C for 90% (v/v) EtOH to achieve comparable yields, which can be industrially less favorable due to increased energy consumption and physical equipment stress. The 0.832 g NaOH/g PET ratio demonstrates a desirable performance, and therefore, exploring a slightly lower concentration like 0.624 g NaOH/g PET becomes a logical next step to potentially reduce chemical usage further without significantly compromising yield or requiring excessive temperatures. Thus, both the 0.624 and 0.832 g NaOH/g PET ratios are preferable as they strike a balance between efficient chemical consumption and less harsh operating conditions. More specifically, these conditions with 90% (v/v) EtOH at 80–90 °C provide an effective compromise, offering a high yield under relatively mild and practical conditions.

#### 3.1.2 Effect of Treatment Time

Following the findings from the DoE experiments, a time-dependent study was conducted to optimize the process. The experimental conditions are summarized in Table 4. Initially, a reaction time of 60 min was found to be sufficient for near-complete hydrolysis of PET at 90 °C using 90% (v/v) EtOH and 0.832 g NaOH/g PET. Building on this, additional experiments were performed to assess the process performance at a lower temperature of 80 °C and NaOH loading of 0.624 g NaOH/g PET. The study also explored the impact of shortening the reaction time from 60 min to 40 and 20 min. These modifications aim to enhance the economic and practical viability of the process by contributing to reduced energy and chemical inputs, which is essential for scaling up to industrial applications.

A consistent trend was observed across the experiments, where higher temperatures (90 °C) consistently yielded higher hydrolysis efficiency than reactions performed at 80 °C, across all reaction times (Figure 6).

**Figure 6:**
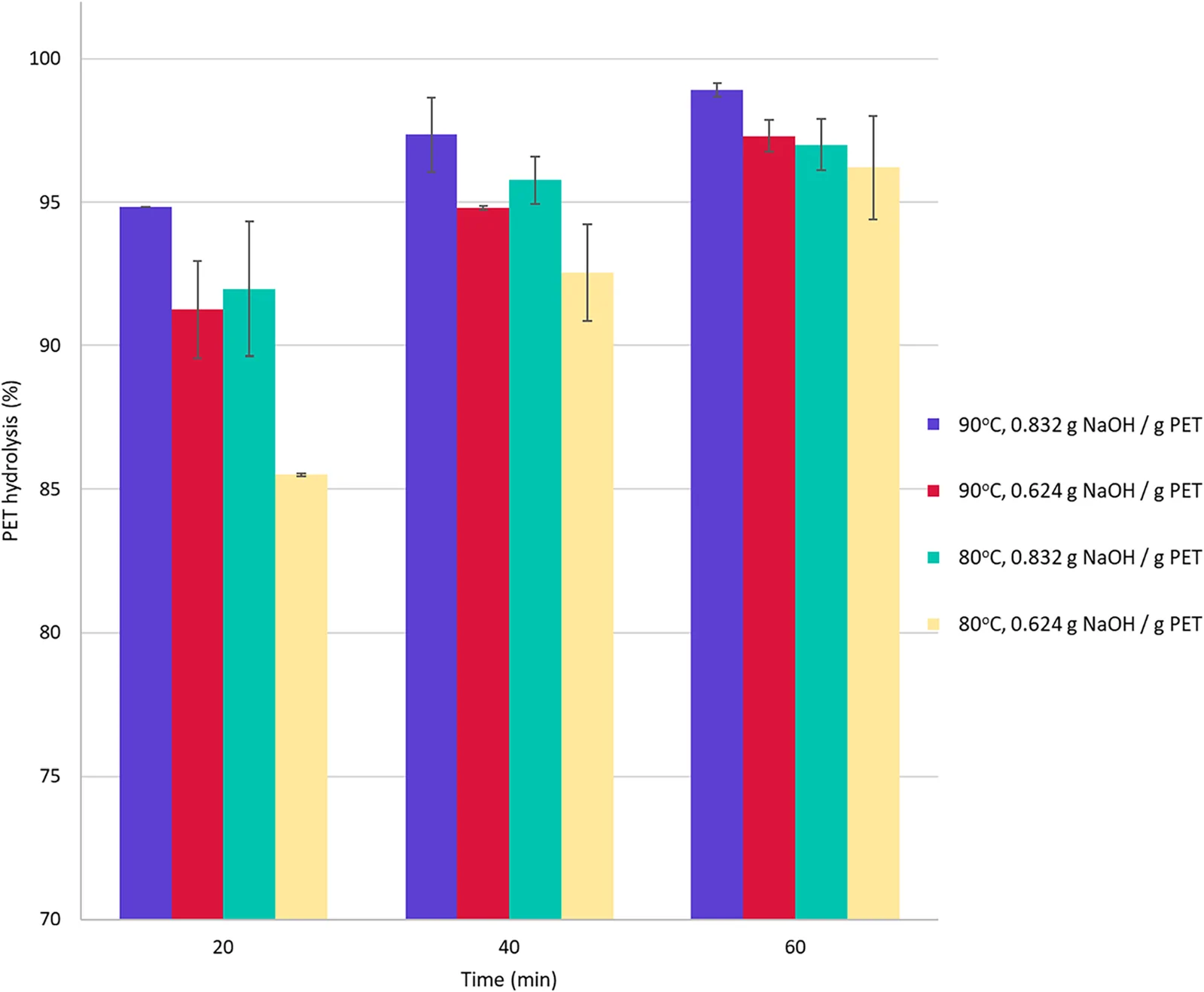
PET hydrolysis yields at reaction times from 20 to 60 min at different sets of conditions.

Additionally, increased NaOH concentration led to improved hydrolysis performance across all of the tested reaction times. Finally, longer reaction times generally resulted in higher yields. Despite these variations, all tested conditions achieved high hydrolysis efficiencies exceeding 91%, with the exception of the mildest conditions tested, *i.e*., 20 min at 80 °C and 0.624 g NaOH/g PET, reaching 85.5% and thus underscoring the robustness of the process. Notably, under a 0.832 g NaOH/g PET ratio, >95% hydrolysis was achieved at both 80 and 90 °C after just 40 min. Reducing the reaction time to 20 min slightly decreased the yields to 94.8% and 92.0% at 90 and 80 °C, respectively. The lowest yield recorded under the mildest tested conditions (0.624 g NaOH/g PET, 20 min) reached 91.3% at 90 °C and 85.5% at 80 °C, still indicating a very high depolymerization at the higher temperature.

These findings highlight the efficiency and flexibility of the HHeAA process, demonstrating that even under reduced temperature, lower chemical loading, and short treatment times, high hydrolysis yields can still be achieved, making the method well-suited for large-scale applications.

### 3.2 Experiments with PET Bottle

#### 3.2.1 Effect of Treatment Time

Following the experiments with PET powder, the HHeAA process was applied to real-life samples using PET water bottles with an estimated crystallinity of 40%. The results, summarized in Figure 7, reveal differences in hydrolysis behavior compared to the PET powder.

**Figure 7:**
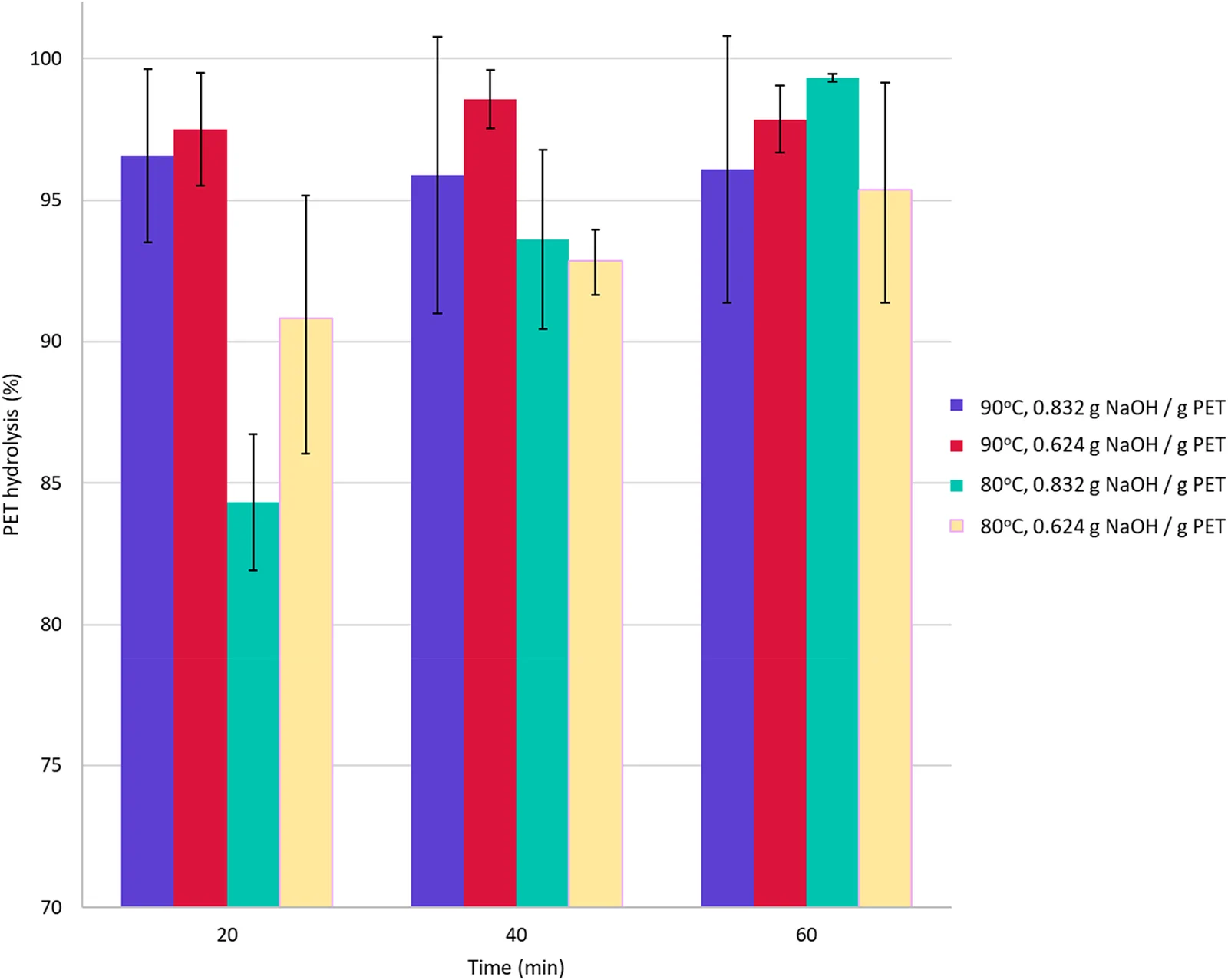
Hydrolysis yields for PET bottles at reaction times from 20 to 60 min at different sets of conditions.

The same experimental conditions were applied, but the previously observed trends with PET powder, particularly the influence of the NaOH concentration and reaction time, were not consistently replicated. The only consistent variable influencing hydrolysis efficiency was temperature, with higher values generally leading to improved outcomes, with a notable exception at 60 min with 0.832 g NaOH/g PET ratio where a slightly higher hydrolysis was observed at 80 °C.

Interestingly, better results were obtained at the shorter reaction time of 20 min with the lower NaOH concentration of 0.624 g NaOH/g PET, on PET bottles. This was observed, not only when compared to PET powder treated at the same conditions but also when compared to PET bottles treated with the higher g NaOH/g PET ratio. Reaction time within the 20–60 min range had negligible influence on the hydrolysis yield when the treatment took place at 90 °C. The highest hydrolysis results observed were 98.6% at 90 °C, 40 min, and 0.624 g NaOH/g PET, and 99.3% at 80 °C, 60 min, and 0.832 g NaOH/g PET. For short-duration treatment of 20 min, the best yield was 97.5%, which was achieved at 90 °C with 0.624 g NaOH/g PET.

Based on these results, the conditions of 90 °C, 20 min, and a g NaOH/g PET ratio of 0.624 were selected for studying the effect of LSR, as it provided an optimal balance between efficiency, energy use, and chemical consumption.

#### 3.2.2 Liquid-to-Solid Ratio (LSR) Study

To evaluate the scalability of the HHeAA process, experiments were carried out with 11.43 and 16.00 g of PET bottle pieces in 80 mL of reaction solution, corresponding to LSRs of 7 and 5, respectively. After the reaction, the slurry was found to be too viscous for efficient vacuum filtration and retained excess liquid. For this reason, the reaction solution and a subsequent water wash (400 mL) were combined into a single filtrate for downstream processing. This shortcoming in the downstream processing could be resolved by using a press filter in an industrial setup, which is more efficient than vacuum filtration for this type of samples. This will also allow for the separation of the solid Na_2_TPA and the reaction liquid, which contains EG. The solid Na_2_TPA could be additionally washed to improve the separation of TPA and EG.

The remaining solids were washed with distilled water, dried overnight in an oven, and used to calculate the hydrolysis yield gravimetrically. Both LSR conditions resulted in high PET hydrolysis (84.4%) (Figure 8).

**Figure 8:**
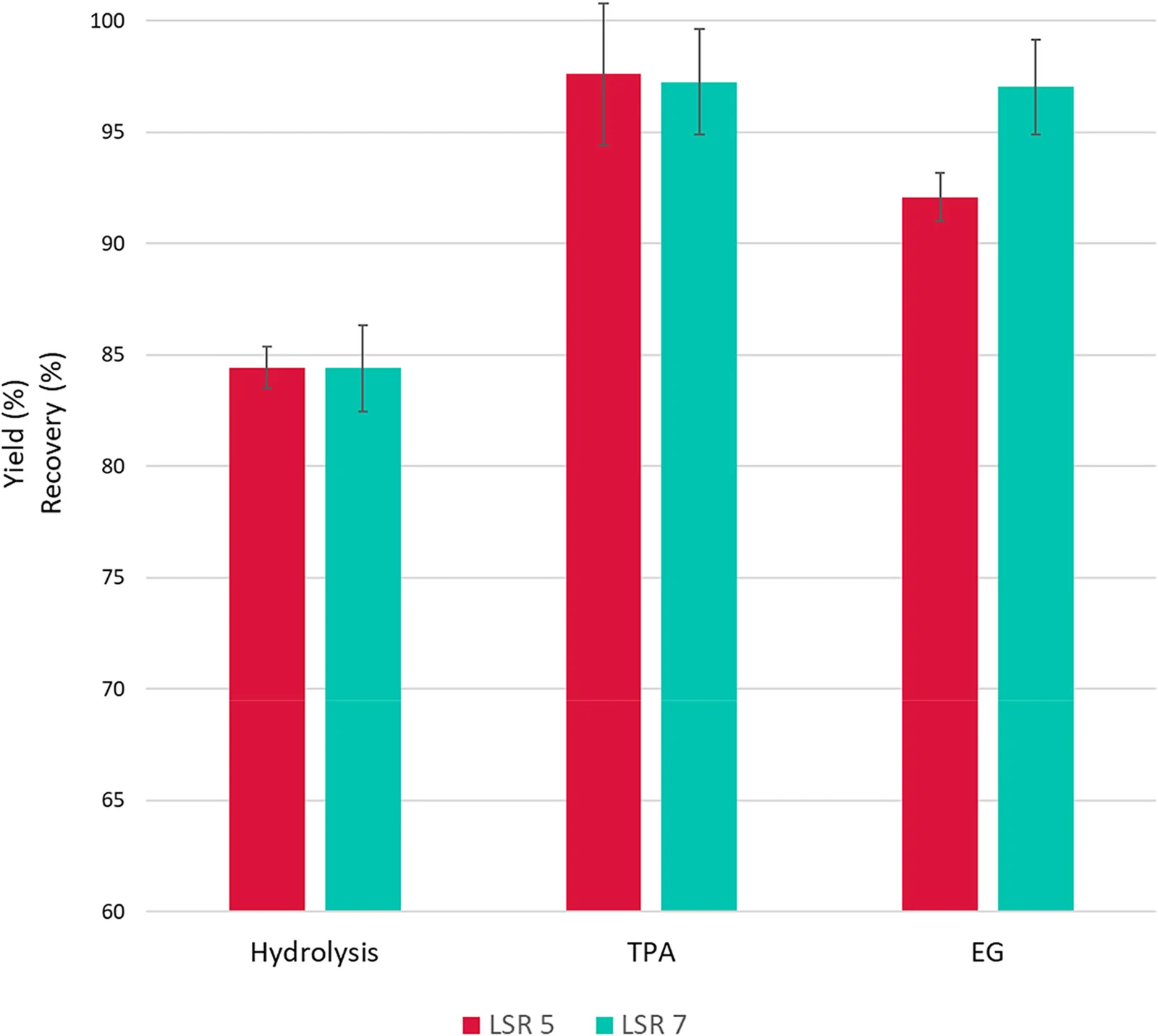
Hydrolysis yields and TPA and EG recovery for experiments with an LSR of 7 and 5.

The filtrate was then analyzed for TPA and EG recovery. Despite the challenges in recovering the reaction liquid, the combined filtrates enabled an accurate mass balance. EG recovery was 97.0% (LSR 7) and 92.1% (LSR 5) with respect to the initial EG, while TPA recovery reached 97.3 and 97.6% of the initial TPA, respectively (Figure 8).

Ash analysis confirmed the absence of inorganic impurities, particularly Na_2_SO_4_, indicating a remarkably high recovery of TPA (LSR = 5).

To verify the complete hydrolysis of PET into its monomers, the isolated TPA powder was analyzed by HPLC to check the possible presence of BHET or mono­(2-hydroxyethyl) terephthalate (MHET). In HPLC analysis, the MHET peak typically elutes between TPA and BHET, with a retention time closer to the latter.^39^ As shown in Figure 9, the absorbance of both samples at 242 nm showed no presence of BHET, or MHET, compared with a standard solution of TPA/BHET.

**Figure 9:**
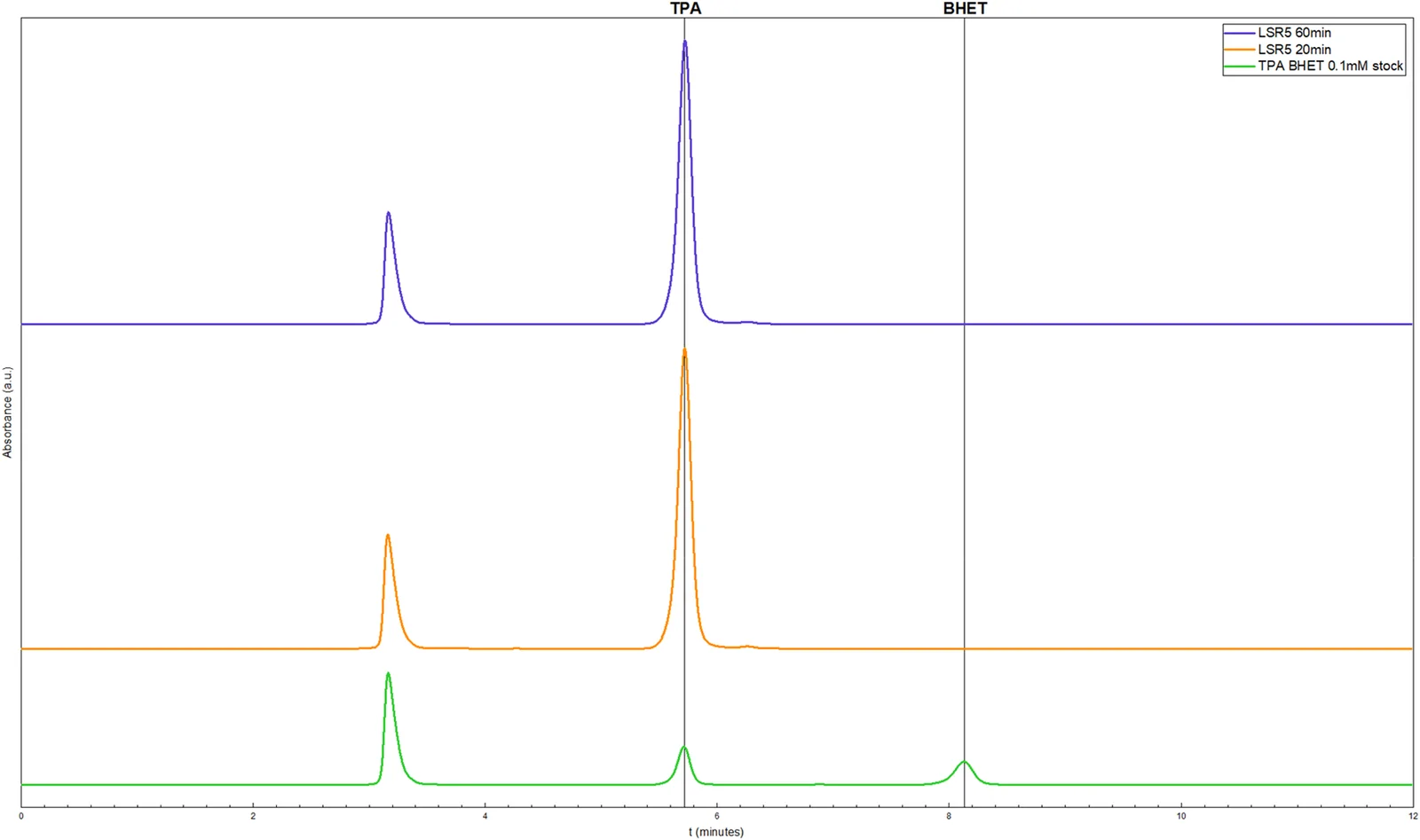
Stacked UV–Vis chromatograms (λ = 242 nm) of TPA samples from LSR 5 at 20 and 60 min, compared to a stock solution of PET/BHET, 0.1 mM.

To further ensure no EG was trapped inside the TPA formation during the acidification step, the isolated powder was incubated overnight in water, which was then analyzed for EG presence. As shown in Figure 10, EG is absent from both samples indicating no residual contamination during the process steps.

**Figure 10:**
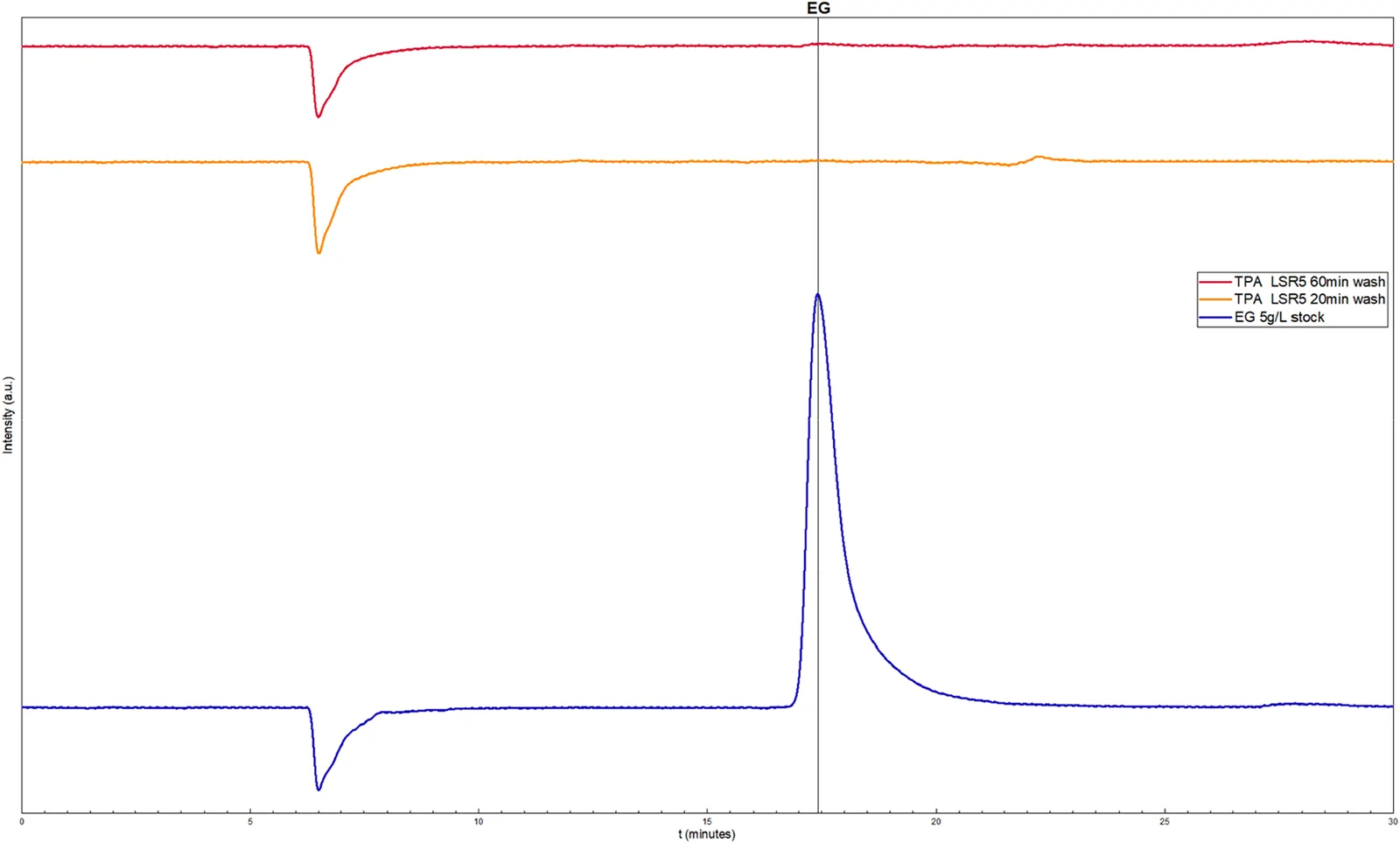
Stacked RI chromatograms of water used to wash TPA samples from LSR 5 at 20 and 60 min, compared to 5 g/L EG stock solution.

^1^H NMR spectroscopy showed the characteristic signal for TPA at 8.0 ppm (Figure 11).^40^ A small signal at 3.4 ppm was observed. That peak could be attributed to both water and EG.^41,42^ Since HPLC analysis proved the absence of EG in the recovered crystals, this NMR signal is assigned to adventitious water.^40^

**Figure 11:**
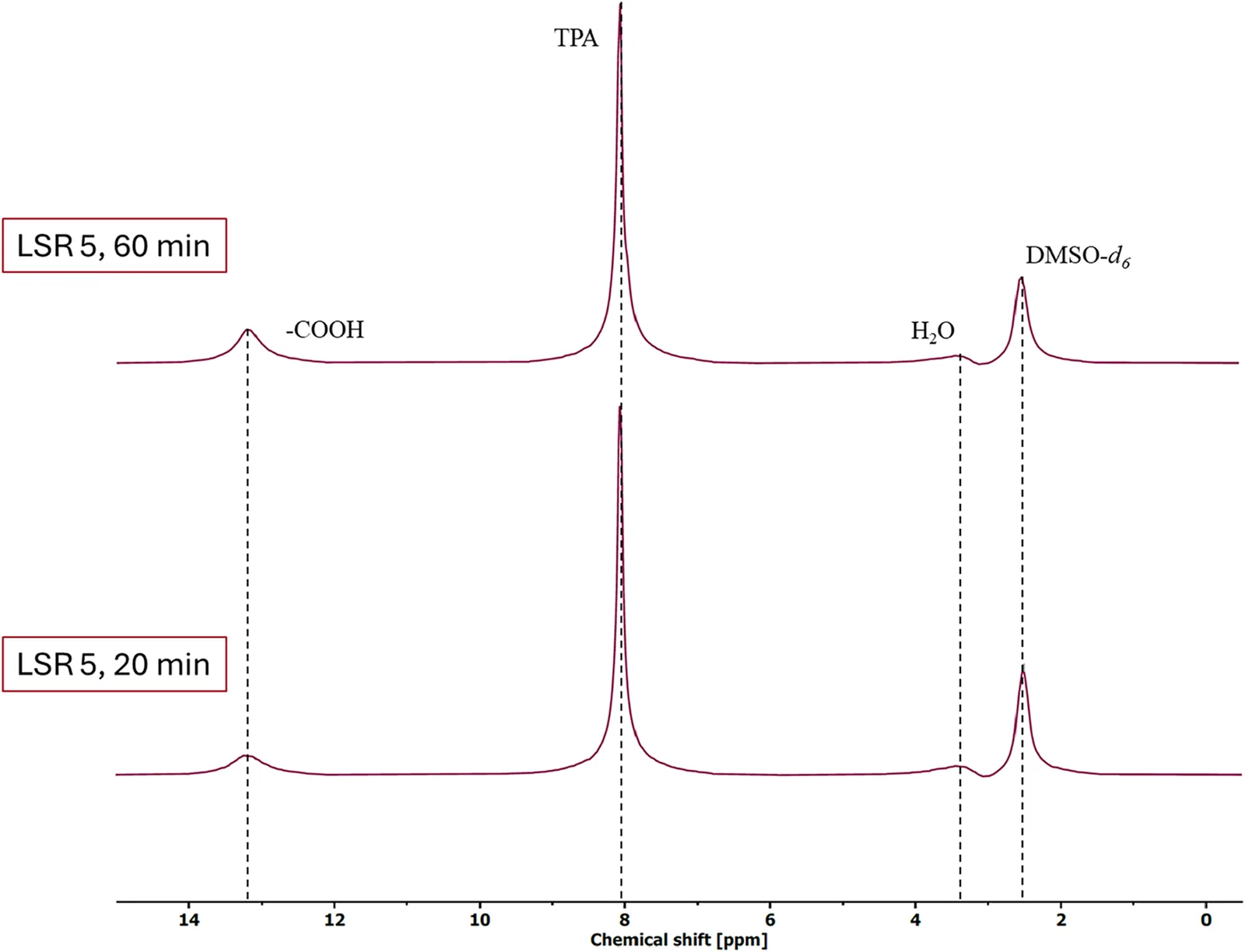
^1^H NMR spectra of the recovered TPA solids.

To further improve hydrolysis efficiency, additional trials were conducted using LSR 5 conditions at extended reaction times of 30, 40, and 60 min. Hydrolysis yield increased progressively, reaching 95.8% at 60 min of reaction time (Figure 12).

**Figure 12:**
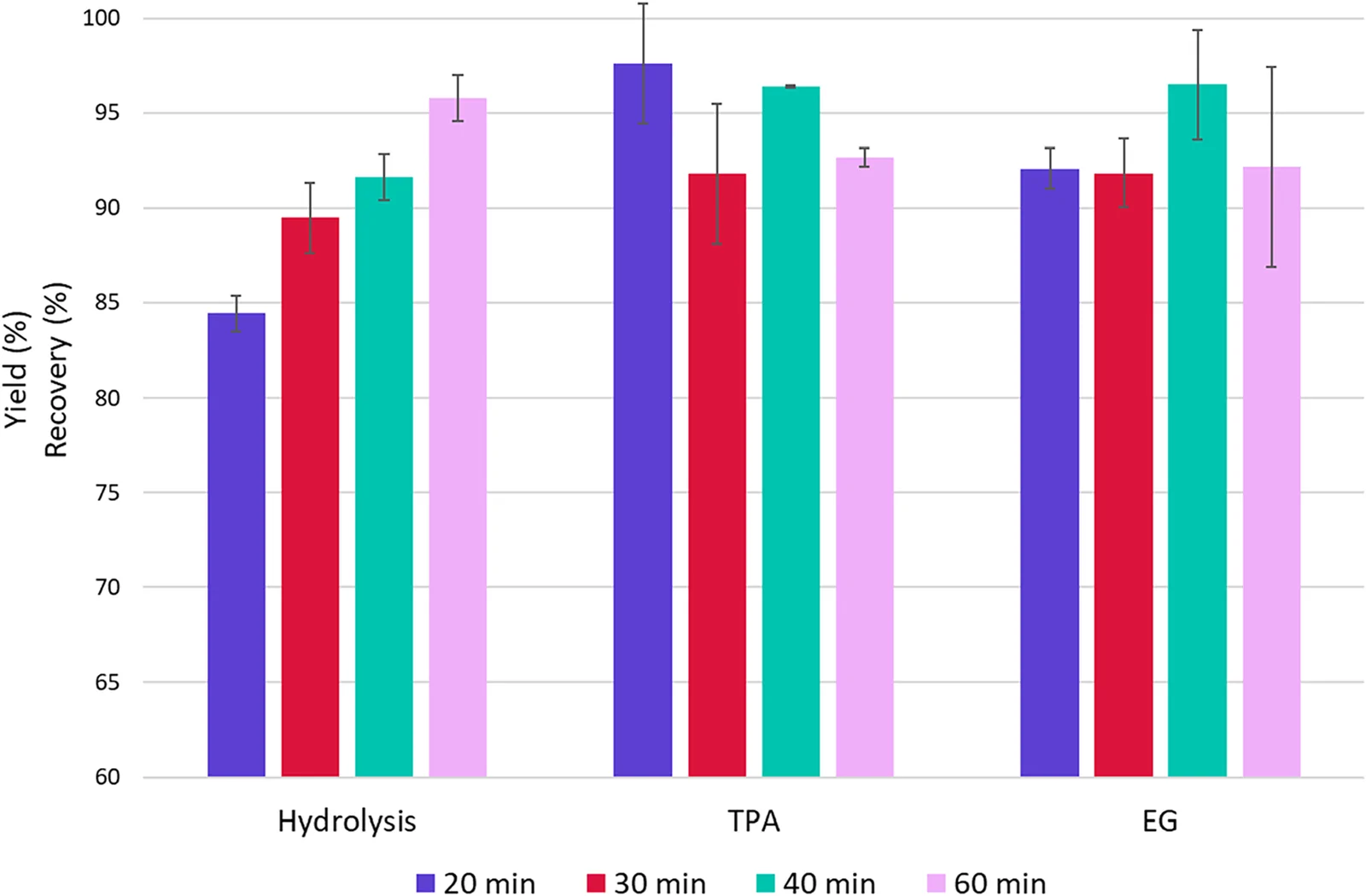
Hydrolysis yield and TPA and EG recovery yields for LSR 5 at different reaction times.

Notably, the improvement from 20 to 30 min is more significant compared to the one from 30 to 40 min. The TPA and EG recovery yields were consistently high, ranging from 91.8% (30 min) to 97.6% (20 min), and 91.8% (30 min) to 92.2% (60 min), respectively (Figure 12). The HPLC and ^1^H NMR analysis of the recovered TPA showed no EG contamination between a 20 and 60 min reaction, and ash content remained negligible.

Regarding the downstream recovery of the solvent, the large boiling point difference between ethanol (78 °C) and ethylene glycol (197 °C) allows for efficient separation *via* distillation. Notably, the literature on extractive distillation demonstrates that ethylene glycol is an effective entrainer for breaking the ethanol–water azeotrope, facilitating the production of anhydrous ethanol.^43^ This suggests a potential synergistic effect where the reaction product (EG) could assist in the dehydration of the recovered solvent. Techno-economic assessments (TEA) of similar organosolv-based biorefineries estimate that solvent recovery to a purity of 99.5% w/w is feasible, by utilizing Mechanical Vapor Recompression (MVR) evaporators to minimize thermal demand.^44^ However, a specialized TEA for the HHeAA process would be needed to determine the overall performance of the scaled process.

To elucidate the gradual progressing of depolymerization on the macroscopic bottle flakes, surface morphology was examined *via* SEM (Figure 13). The images reveal a distinct morphological evolution driven by the semicrystalline and oriented nature of the PET feedstock.

**Figure 13:**
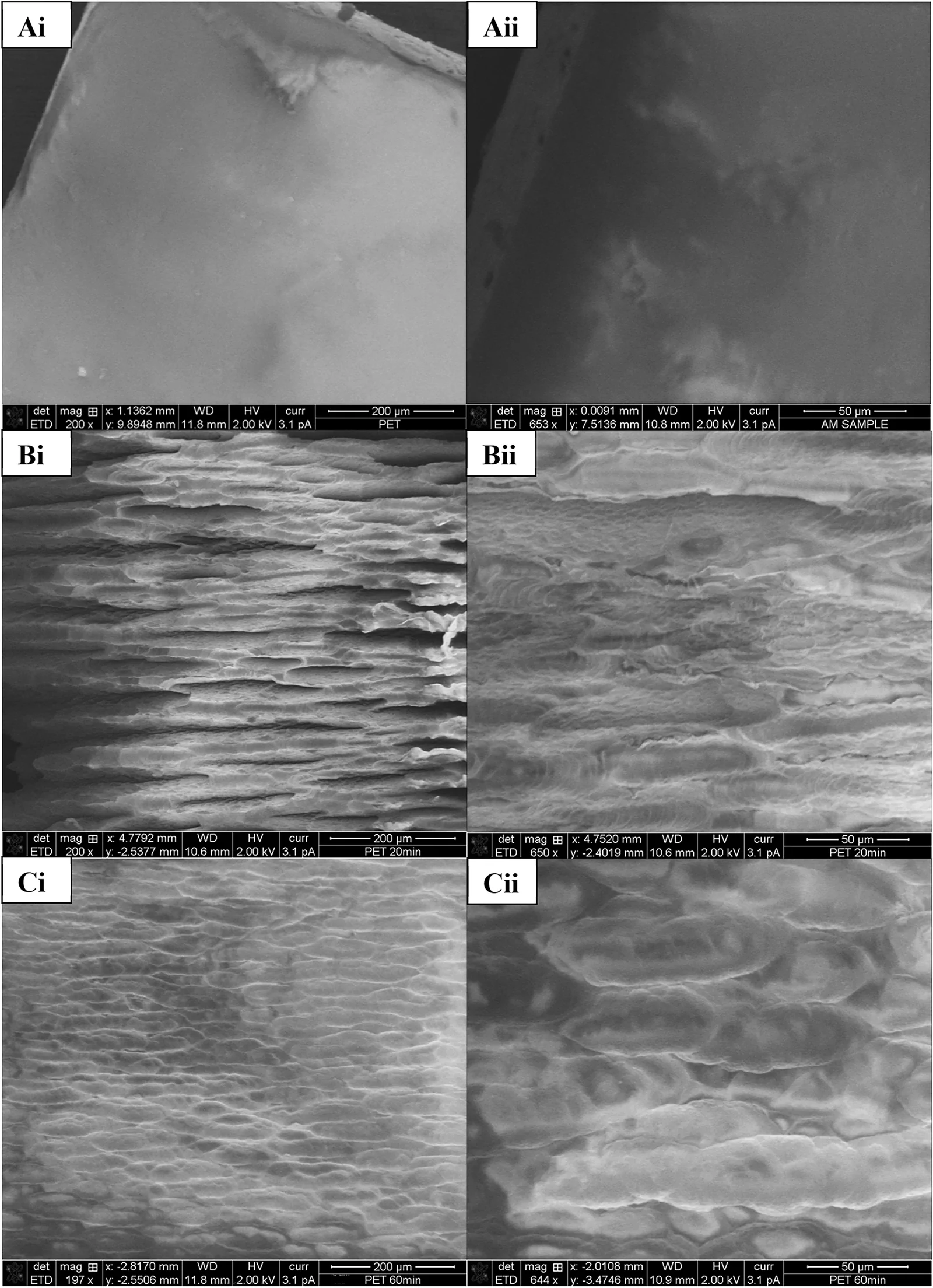
SEM micrographs of PET bottle flakes treated with the HHeAA process. (A) Untreated bottle, showing a smooth surface with no visible damages. (B) After 20 min, showing deep directional striations and grooves caused by the preferential degradation of amorphous regions between oriented polymer fibrils. (C) After 60 min, showing a nodular “cobblestone” morphology, indicative of exposed resistant crystalline domains following extensive surface erosion. There are two magnifications for each sample: (i) ∼200x and (ii) ∼650x.

After 20 min of reaction, the surface of the unreacted PET is characterized by deep, directional striations and fibrous grooves (Figure 13B). This topology results probably from the preferential hydrolytic attack on the more accessible amorphous regions located along and between the aligned polymer fibrils, highlighting the biaxial orientation of the original water bottle.^45,46^ As the reaction progresses to 60 min (Figure 13C), the morphology transitions to a pronounced nodular or “cobblestone” structure. These protruding nodules likely represent the highly crystalline domains (spherulites) of the polymer, which are more resistant to alkaline attacks than the surrounding amorphous matrix.^47–49^ This progression confirms that the HHeAA process operates via a surface erosion mechanism, where ethanol-induced swelling is critical to exposing the internal amorphous regions of the dense, oriented polymer matrix.^35^ This morphological restriction also explains the differences in the hydrolysis efficiency observed between PET bottles and powder. Powder possesses a high initial surface-to-volume ratio, enabling an immediate reaction, while the compact bottle flakes rely on the gradual solvent-driven surface treatment for efficient depolymerization.

These results demonstrate that the HHeAA process can effectively treat waste plastic bottles, maintaining high efficiency at low LSR and reduced NaOH concentrations, such as 0.624 g NaOH/g PET. Compared to the current state of chemical recycling technology, HHeAA offers significant advantages in operation simplicity and sustainability.

As shown in Table 7, most traditional alkaline hydrolysis methods require higher temperatures, longer reaction times, and higher LSRs, making them less favorable for industrial implementation. For example, complete hydrolysis (98%) using water as the sole solvent required 200 °C for 1 h, while another study achieved an 84% yield by increasing LSR to 130 to compensate for a lower temperature of 95 °C.^22,50^ However, such high LSRs are impractical for large-scale use and pose a challenge for the economic viability of the process.^30^ The use of catalysts can enable lower temperature hydrolysis but still often requires longer reaction times or higher NaOH concentrations, as seen with the TBHDPB (tributylhexadecylphosphonium bromide quaternary salt).^22,51^

**Table 7:** Overview of the Performance of Hydrolysis and Glycolysis Methods for PET

method	PET	LSR	solvent	alkaline catalyst	alkaline catalyst/PET (g/g)	catalyst	temperature	time	reaction yield (%)	refs
hydrolysis	flakes from bottles	10	H_2_O	NaOH	0.45		200 °C	1 h	98%	^22^
							120 °C	7 h	33%	
hydrolysis	flakes from bottles	10	H_2_O				250 °C	90 min	91.6%	^54^
hydrolysis	flakes from bottles	130	H_2_O	NaOH	6.5		95 °C	1 h	84%	^50^
hydrolysis	particles from trays	10	H_2_O	NaOH	0.65 g/g		100 °C	4 h	34%	^51^
						TBHDPB (0.1 wt %)			90%	
hydrolysis	particles from trays, films, bottles	50	60% EtOH	NaOH	2.5 g/g		80 °C	20 min	95.2%	^24^
ethanol-assisted hydrolysis	pellets from bottles	25	90% EtOH	KOH	5 g/g		80 °C	30 min	97.6%	^23^
					1.25 g/g			1 h	95.3%	
					0.5 g/g				73.9%	
hydrolysis	chips from bottles	2.86	H_2_O				200 °C	24 h	97.7%	^53^
							210 °C	8 h	83%	
glycolysis			EG						76%	
glycolysis	powder from bottles	N.A.	EG			ZnMn_2_O_4_ (1 wt %)	260 °C	1 h	92.2%	^55^
glycolysis	flakes from bottles	N.A.	EG			Co(C_2_H_3_O_2_)_2_ (0.002 mol)	190	1.5 h	98.8%	^57^
								1 h	85.7%	
									0%	
						Co(C_2_H_3_O_2_)_2_ (0.002 mol)	110%		0%	
							130 °C		0%	
							150 °C		0.5%	
							170 °C		11.8%	
ethanol-assisted hydrolysis	pristine powder	20	90% EtOH	NaOH	0.624 g/g		90 °C	20 min	91.3%	current study
								1 h	97.3%	
	pieces from bottles	5						20 min	84.4%	
								1 h	95.8%	

The HHeAA process builds upon previous research into ethanol-assisted hydrolysis. A study in 2021, using potassium hydroxide in various alcohols (methanol, ethanol, iso-propanol, and tert-butanol) in the absence of water, reported only 16% PET hydrolysis irrespectively of the alcohol used, suggesting that they function as solvents, not reactants.^52^ Meanwhile, the presence of water is critical, as it increases the solubility of the alkaline catalyst and enhances reactivity.^52^ While other studies have shown the benefits of using ethanol, they often require much higher LSRs or alkaline catalysts concentrations to achieve high yields, making them economically unviable.^23,24^ For instance, a 95.2% yield was achieved with 60% ethanol, but at a high LSR of 50, resulting in a NaOH/PET ratio of 2.5 g/g.^24^ In contrast, our process achieves near-complete hydrolysis (95.8%) under mild conditions and with one of the lowest reported NaOH/PET ratios of 0.624 g/g.

While compared to other chemical recycling methods such as neutral hydrolysis and glycolysis, the HHeAA process proves superior in terms of reaction conditions. Neutral hydrolysis, while effective, requires significantly longer reaction times at high temperatures, which is not economically feasible for large-scale use.^53,54^ Glycolysis, even with catalysts, often requires severe conditions, such as high temperatures (up to 260 °C) and long reaction times to reach high yields.^55,56^ Overall, our data suggest that the HHeAA process demonstrates substantial improvements over conventional hydrolysis and glycolysis technologies, enabling complete hydrolysis at mild temperatures, short reaction times, and a significantly lower NaOH loading. This advancement has significant implications for the economic and environmental feasibility of PET chemical recycling and positions the HHeAA process as a leading candidate for industrial adoption.

## 4 Conclusions

This study presents significant advancements in the development and optimization of the HHeAA process, originally designed for textile recycling, by successfully adapting it to PET bottle depolymerization. The process operates under markedly milder conditions. This study establishes the HHeAA process as a superior alternative to conventional chemical recycling methods by explicitly addressing the limitations of high chemical consumption and severe reaction conditions. Quantitatively, this work demonstrates near-complete depolymerization (96%), by operating at low temperatures (90 °C), and reduced NaOH (0.624 g NaOH/g PET) compared to what is commonly reported in the literature, representing a significant reduction in caustic consumption compared to previous high-yield ethanol-assisted protocols, and one of the lowest reported LSR of 5, compared to conventional methods reported in the literature. These optimizations can substantially enhance the economic and environmental viability of the process for industrial-scale implementation and make HHeAA more relevant for potential implementation compared to previous studies.

Improvements were also achieved in the downstream processing. This was evident by the very high purity of the recovered TPA, as confirmed by a combination of analytical techniques (HPLC, ^1^H NMR, and ash analysis), with no EG contamination. Additionally, this study is one of the few that followed and demonstrated the fate of EG and analyzed quantitatively its presence in the fractions. A high recovery rate of EG was achieved, further validating the process efficiency. Furthermore, a significant reduction in NaOH usage, without compromising hydrolysis yield, represents a critical advancement toward cost-effective scalability.

Overall, the enhanced HHeAA process successfully addresses key limitations of current chemical recycling technologies. Its ability to operate under mild conditions, process high-solid-load systems, and achieve a high product recovery yield with reduced chemical input makes it a promising candidate for future industrial applications in sustainable PET waste recycling.

## Supplementary Material



## Notes

DSC analysis of the pristine PET and the PET bottles used for the experiments (PDF)
