# Supplementary material for: Efficient Chemical Recycling of Polyester in Plastic Waste: A Heated High-Ethanol Alkaline Aqueous Process
Source: Org Process Res Dev. 2026 Feb 10;30(2):384–400. doi: 10.1021/acs.oprd.5c00386 (PMC12930499; doi:10.1021/acs.oprd.5c00386)
Supplement: Supplementary file 1 [file op5c00386_si_001.pdf]

Efficient Chemical Recycling of Polyester in Plastic Waste: A Heated High-Ethanol Alkaline Aqueous Process

Kalliopi Elli Pavlopoulou[†], Vincenzo Ianniello[‡], Kateřina Hrůzová[†], Theo A. Tervoort[‡], Heiko Lange[†,§,‖], Ulrika Rova[†], Paul Christakopoulos[†], Leonidas Matsakas[,†]*

[†] Biochemical Process Engineering, Dept. of Civil, Environmental and Natural Resources Engineering, Luleå University of Technology, SE-971 87 Luleå, Sweden

[‡] Department of Materials, ETH Zurich, Vladimir-Prelog-Weg 5, 8093, Zurich, Switzerland

[§] Department of Earth and Environmental Sciences, University of Milano-Bicocca, Piazza della Scienza 1, 20126 Milan, Italy

[‖] NBFC – National Biodiversity Future Center, 90133 Palermo, Italy

[*] Corresponding author. Email address: Leonidas Matsakas, Department of Civil, Environmental and Natural Resources Engineering, SE-971 87 Luleå Sweden, leonidas.matsakas@ltu.se, tel.: +46 (0) 920 493043

Supporting Information: DSC analysis of the pristine PET and the PET bottles used for the experiments

This supplementary information provides additional data related to the thermal characterization of pristine and bottle-grade polyethylene terephthalate (PET) polymer samples. Differential Scanning Calorimetry (DSC) was employed to determine key thermal properties, including melting enthalpy (ΔH_m) and crystallinity (X_c), for both sample types.

Figure S 1 presents the DSC thermograms, illustrating the thermal behavior of the pristine and bottle-grade polymer samples over a range of temperatures. Table S 1 summarizes the quantitative data extracted from these DSC analyses, specifically reporting the calculated ΔH_m, crystallization enthalpy (ΔH_c), and X_c values. These data support the discussions on the material properties within the main manuscript.

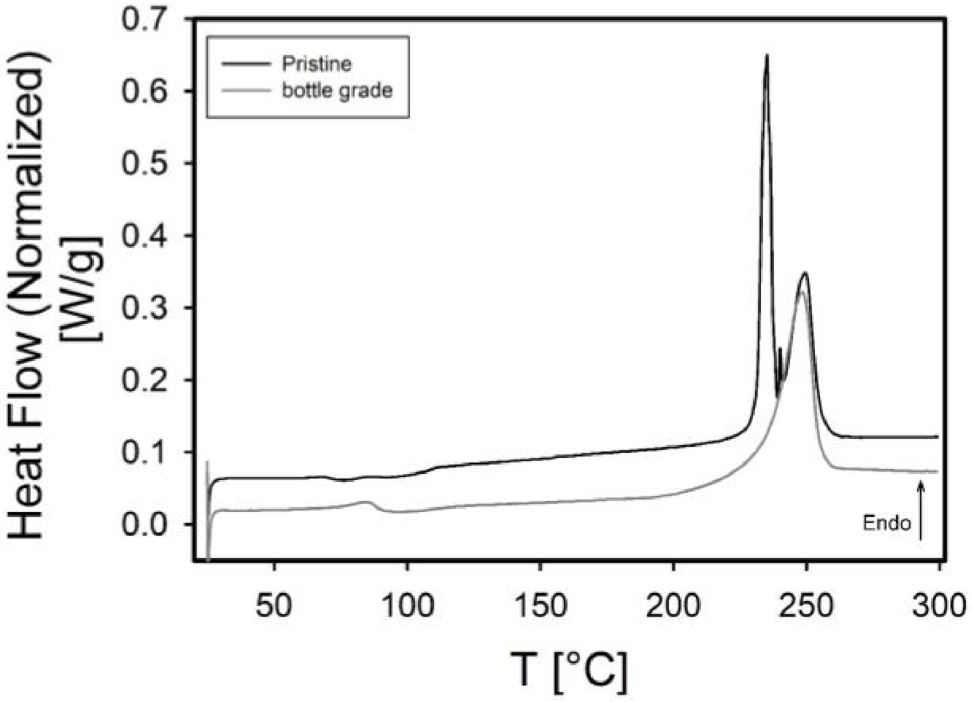

Figure S 1. DSC analysis of pristine and bottle grade polymer samples.

Table S 1.Thermal properties and crystallinity of pristine and bottle grade polymer samples.

**Sample**	**ΔH_m [J/g]**	**ΔH_c [J/g]**	**c**
Pristine	63.9	/	0.46
Bottle grade	55.7	/	0.40
